# Meta‐Analysis of Cost‐Effectiveness

**DOI:** 10.1002/sim.70352

**Published:** 2026-03-18

**Authors:** Heejung Bang, Hongwei Zhao

**Affiliations:** ^1^ Division of Biostatistics, Department of Public Health Sciences University of California, Davis Davis California USA; ^2^ Division of Occupational and Environmental Health, School of Medicine University of Utah Salt Lake City Utah USA

**Keywords:** bootstrap wedge, cost‐effectiveness analysis, cost‐effectiveness plane, health economics, incremental cost‐effectiveness ratio (ICER), systematic review

## Abstract

Systematic review and meta‐analysis are widely accepted approaches for evaluating treatment effectiveness. Meta‐analysis generally addresses statistical aspects of systematic reviews, such as the pooling of treatment effect sizes, assessment of heterogeneity, and statistical inference. To complement treatment effectiveness, cost‐effectiveness is often conducted to encompass both clinical and economic perspectives. However, there are few statistical methods proposed for meta‐analyses of cost‐effectiveness, and none is used widely. In fact, meta‐analysis is currently not encouraged for cost‐effectiveness due to methodological and statistical complexities. In this paper, we propose simple meta‐analytic methods for cost‐effectiveness, which may serve as a starting point for future work. We illustrate the methods using two examples from systematic reviews on wound interventions and mental illness.

## 1 Introduction

Every year, numerous meta‐analyses are published. In January 2025, Google Scholar found over 5 million entries when “meta‐analysis” was searched. The most important and relevant meta‐analyses are on treatment effectiveness in randomized controlled trials (RCTs), although observational studies or non‐comparative measures such as disease prevalence can be meta‐analyzed. Frequently, after multiple RCTs with comparable parameters and settings (e.g., Population, Intervention, Comparison, Outcomes [PICO]) are published, a meta‐analysis is a natural next step. When conducting an RCT whose primary aim is the treatment effectiveness or efficacy analysis (EA) of an intervention versus a comparator, cost‐effectiveness analysis (CEA) can be concurrently or subsequently performed as a second aim.

The incremental cost‐effectiveness ratio (ICER) serves as the most commonly employed metric in CEA along with quality‐adjusted life years (QALY) as a clinical outcome for an effectiveness measure [1, 2, 3]. However, very few meta‐analyses have been conducted for CEA; some systematic or literature reviews exist in the literature. Some have offered reasons for this paucity [4]. Meta‐analysis is a highly “statistical” process that is often performed concurrently with or after systematic review. Key methodological or statistical barriers in CEA, which may differ from EA are that: (1) it is challenging to derive an overall (pooled) summary measure of ICER as a *ratio* statistic, a scalar measure combining two‐dimensional information conveyed for cost and effectiveness, and (2) summary statistics generally needed as inputs for meta‐analysis (e.g., sample size [*N*], effect size [ES], and standard error [SE] or its variant for a variability measure related to ICER) are not always reported or derivable from original CEA publications. Furthermore, CEA based on mathematical or probabilistic modeling instead of *observed* data is a popular and legitimate line of research, but it normally does not provide the actual sample size of patients and statistical variability measures. Nowadays, CEA based on empirical data—in parallel with EA—is increasingly conducted; statistical methods to facilitate meta‐analyses for CEA can be useful so that meta‐analyses for EA and CEA may be presented side by side whenever feasible and justified.

Currently, most systematic reviews of economic evaluations are based on descriptive and qualitative summaries of evidence, including Cochrane and other guidelines [5, 6, 7]. Crespo et al. [8] proposed a method for meta‐analysis of CEA studies using simulated data. Their method is based on incremental net benefit (INB) and Copula, which naturally entail willingness‐to‐pay (WTP) thresholds, which vary or can be subjective, and parametric distributional assumptions, with difficulty in choosing the right Copula. Bagepally et al. [6] provided guidelines on data harmonization for Crespo's meta‐analytic methods, and explained that data imputation (e.g., variance of INB) or reduction in the number of studies to be included can be needed in the real‐world implementations. Tricco et al. [9] and Dewa et al. [10], among others, published systematic reviews of CEA focused on clinical topics but did not attempt meta‐analysis. Conombo et al. [11], Bagepally et al. [6], and Veettil et al. [7], among others, used a Forest plot—a standard graphic tool in meta‐analysis—to display the point estimates of INB of individual studies together. From the mathematical perspective, INB is a rearrangement of ICER via a difference instead of a ratio, so that the statistical inferences of INB (with fixed WTP) and associated meta‐analyses can be done in the traditional framework [7, 12].

In this paper, we propose meta‐analytic methods based on the ICER for CEA as a natural extension of standard meta‐analysis for EA. We separately pool the cost difference (numerator) and the effectiveness difference (denominator) and compute their corresponding confidence intervals (CIs). Then we compute the ICER with the pooled mean cost and the pooled mean effectiveness, and a CI of the ICER. Final results—which include individual cost and effectiveness, pooled cost and effectiveness, and their CIs, and a pooled ICER—are displayed together in the CE plane. In Section 2, we outline statistical methods. In Section 3, we present data analyses. We conclude with discussions relevant and unique to CEA.

## 2 Statistical Methods

A conventional summary measure for CEA is the ICER, defined as

ICER=ΔC/ΔE

where Δ*C* = *C*1 − *C*0 represents the difference in mean costs between an experimental treatment group (1) and a control treatment group (0), and Δ*E* = *E*1 − *E*0 is the corresponding difference in mean effectiveness. The ICER can be interpreted as the extra or *incremental* cost incurred per 1 unit increase in effectiveness (e.g., additional cost for an additional 1 year of life if *E* is measured in life years), and it is a slope in the CE plane. ICER serves as a fundamental, quantitative decision aid in the CEA context. A measure for *E* that is commonly used to inform policy decisions (e.g., whether to pay for a drug) is the QALY, usually a continuous variable or based on survival data, while cost is expressed in monetary units and should be converted to a common metric before meta‐analysis (e.g., United States dollar [USD] in 2024).

If effectiveness is a binary variable, its mean can be interpreted as a probability, so that ICER can be interpreted as the additional cost incurred for one more success or % increase in the event rate (or decrease in risk). For a count outcome, cases averted can be employed as a clinical outcome [1]. Also, the mean difference (MD) above can be replaced by the standardized mean difference (SMD) if original metrics are in disparate scales (e.g., various depression severity scales as effectiveness), where MD and SMD abbreviations are commonly used in meta‐analysis literature and software packages. Of note, in this paper, we use estimand/parameter and estimator/estimate interchangeably when the context is clear for efficient communication. The ICER parameter can be estimated by plug‐in estimators of the sample means. Also, we use the arithmetic mean—rather than the median or a log‐transformation—based on the current CEA practice guidelines [2, 3, 13].

We propose to perform a meta‐analysis on CEA by the following procedure. First, we estimate the difference in mean costs (deltaC, or ΔC) by a pooled estimate and a 95% CI for the pooled cost difference. Second, we repeat the process for the effectiveness counterpart (deltaE, Δ*E*). Finally, we calculate the resulting ICER using the ratio of the pooled cost difference and effectiveness difference. We propose to add these results to the CE plane along with individual study‐specific ICER values (strictly speaking, individual deltaC and deltaE) and interpret. We generally avoid pooling ICERs directly since interpretation should take qualitatively different scenarios on the two‐dimensional statistics and four‐quadrant CE plane into account; for example, +1/+1 = 1 versus −1/−1 = 1 and 10/0.0001 versus 10/(−0.0001) can have quite different CE implications despite the same or seemingly close value in it. Moreover, between averages of ratios versus the ratio of averages, there is greater stability by taking the average before taking the ratio. We explain some caveats when we illustrate examples in Sections 3 and 4.

We describe statistical methods for pooling the costs and effectiveness estimates and obtaining their CIs, which address two of the most common scenarios in the CEA literature.

### 2.1 Scenario 1: { *ΔC* , *ΔE* , *N*} Available From Each Study, but SEs Are Not Available

#### 2.1.1 “*Ratio*” Method

This scenario may represent the most common data availability in CEA or can be useful when a variability measure (e.g., standard deviation [SD], SE, variance, or CI) is not reported, reliable, or well defined (e.g., open CI) in original publications. In this scenario, we will use a “Ratio” method based on classical survey sampling for obtaining the pooled cost estimate and the pooled effectiveness estimate, as proposed by Shuster [14, 15]. Shuster called this a “Studies‐at‐Random” approach. Key aspects of this approach are: (1) to treat each study as an independent sample/cluster similar to each person (in simple random sampling) in traditional RCTs; (2) to allow the sample size of each study to be random so that effect size and sample size may be correlated; and (3) that SEs typically needed for traditional meta‐analysis are not required. In practice, condition (3) is attractive as SE is often unavailable in CEA, which is a common barrier, more relevant to model‐based CEA than trial‐based CEA. This approach allows for the inclusion of the maximum number of potentially qualified studies in the meta‐analysis of CE results.

For a general framework for the ratio estimation, let {*Y*_*j*_, *Z*_*j*_; *j* = 1,…,*M*} from *M* studies be an independently and identically distributed (iid) random vector with mean (*μ*_*y*_, *μ*_*z*_), where *j* denotes the *j*‐th study that constitutes the meta‐analysis. We assume that *M* is reasonably large (at least 5–20). The ratio of the sample means

$$\bar{Y}/\bar{Z}=\left(\sum_{j=1}^{M}Y_{j}/M\right)/\left(\sum_{j=1}^{M}Z_{j}/M\right)$$

has been empirically shown to be well approximated as an asymptotic *t*‐distribution with (*M* − 2) degrees of freedom (df) with the population means *μ*_*y*_/*μ*_*z*_ and asymptotic variance based on Taylor's theory [14]

(1) $$V^{2}=\left\{{\left(\sigma_{y}/\mu_{z}\right)}^{2}+{\left(\mu_{y}\sigma_{z}/{\mu_{z}}^{2}\right)}^{2}-2\rho_{y,z}\mu_{y}\sigma_{y}\sigma_{z}/{\mu_{z}}^{3}\right\}/M$$

where *ρ*_*y,z*_ is the correlation coefficient between *Y*_*j*_ and *Z*_*j*_.

Using the “Ratio” method, the pooled cost difference estimator can be obtained as follows:

$$\Delta C_{\text{pooled}}=\sum_{j=1}^{M}N_{j}{\Delta C}_{j}/\sum_{j=1}^{M}N_{j}=\left(\sum_{j=1}^{M}N_{j}{\Delta C}_{j}/M\right)/\left(\sum_{j=1}^{M}N_{j}/M\right)$$

$$=\left(\sum_{j=1}^{M}Y_{j}/M\right)/\left(\sum_{j=1}^{M}Z_{j}/M\right)=\bar{Y}/\bar{Z}\;$$

where *Y*_*j*_ = *N*_*j*_Δ*C*_*j*_ and *Z*_*j*_ = *N*_*j*_ is the total sample size for study *j* where two arms are combined. For obtaining the pooled effectiveness difference, we can simply replace Δ*C*_*j*_ by Δ*E*_*j*_ and repeat the same process for plug‐in estimators. The point estimator for generic $\bar{Y}/\bar{Z}$ can be used for cost first and for effectiveness next. Cost alone and effectiveness alone can be of interest, where the former is comparison of the costs (e.g., for cost minimization) and the latter is a type of EA [2, 3]. Finally, the ICER can be written as the ratio of these two ratios

$$\text{ICER}=\left(\sum N_{j}{\Delta C}_{j}/\sum N_{j}\right)/\left(\sum N_{j}{\Delta E}_{j}/\sum N_{j}\right)$$

where summation is over study index *j*. Owing to additivity, the numerator can be expressed as *∑N*_*j*_Δ*C*_*j*_ */∑N*_*j*_ = (*∑N*_*j*_ *C1*_*j*_ */∑N*_*j*_) − (*∑N*_*j*_ *C0*_*j*_ */∑N*_*j*_), where 1 and 0 denote the treatment indicator; this difference would be the mean cost if all patients received the treatment 1 minus the mean cost if all patients received the treatment 0. The population parameter would be identical to the estimate if all studies in the population were sampled [14]. A consistent estimator of *V* ^2^ in (1) can be obtained by the method of moment estimators for the five population parameters {*μ*_*y*_, *μ*_*z*_, *σ*_*y*_, *σ*_*z*_, *ρ*_*y,z*_} in *V* ^2^ and a 100×(1−α)% CI can be formed by a pooled estimate *±t*_*α*/2_,_*M*−2_ $\hat{V},$ along with *p* value, if desired.

#### 2.1.2 “Ratio” Method Treating Sample Sizes as Constants

We also considered the situation when *N*_*j*_ is assumed to be non‐random or constant. Hunter and Schmidt used it for meta‐analysis on the correlation coefficient [16]. In this case, a variance formula for the pooled cost difference can be written as

$$V^{2}=\sum \left[{N_{j}}^{2}\sigma^{2}\Delta C_{j}\right]/{\left(\sum N_{j}\right)}^{2}=\left[\sum {N_{j}}^{2}\right]/{\left(\sum N_{j}\right)}^{2}\sigma^{2}\Delta C_{j}$$

where $\sigma^{2}\Delta C_{j}$ can be estimated by a sample variance of observed *M ΔC*_*j*_s, and we use *t*_*α*/2_,_*M*−1_ in the construction of a 100 × (1 − *α*)% CI. We can repeat the same process for *E*. Naturally, the point estimates from the two ratio methods are identical.

### 2.2 Scenario 2: { *ΔC* , *ΔE* , SE_*ΔC*_ , SE_*ΔE*_ } Available From Each Study

In this scenario, we consider inverse‐variance weighting (IVW)–based “mainstream” methods for meta‐analysis. We repeat the same procedure for cost and effectiveness (for numerator and denominator) separately, as before. Shuster called this approach “Effect‐at‐Random,” where it is a “weighted average” with weights assumed to be constants [14].

#### 2.2.1 Fixed Effect (FE) IVW Method

Generic formulas for FE estimator based on IVW for a pooled measure ES are written as

$$\bar{\mathrm{ES}}=\left(\sum_{j=1}^{M}w_{j}{\mathrm{ES}}_{j}\right)/\left(\sum_{j=1}^{M}w_{j}\right)$$

where *w*_*j*_ = 1/SE_*j*_^2^, and its variance can be estimated by

$$\hat{V^{2}}\left(\bar{\mathrm{ES}}\right)=1/\sum_{j=1}^{M}w_{j}$$

#### 2.2.2 Random Effect (RE) IVW Method

In a RE model, we assume

(2) $${\mathrm{ES}}_{j}=\text{true}\;\mathrm{ES}+u_{j}+\sigma_{j}\epsilon_{j}$$

where *ϵ*_*j*_ ∼ *N*(0,1) and *u*_*j*_ ∼ *N*(*0*, *τ* ^2^), both iid, and *u* and *ϵ* are independent.

*Q* statistic and *I* ^2^ that assess heterogeneity or inconsistency can be written as: $Q=\left(\sum_{j=1}^{M}w_{j}{\mathrm{ES}}_{j}^{2}\right)-{\left(\sum_{j=1}^{M}w_{j}{\mathrm{ES}}_{j}\right)}^{2}/\left(\sum_{j=1}^{M}w_{j}\right)$ and *I* ^2^ = max{(*Q* − df)/*Q*, 0}, where df = *M* − 1. Then we define a well‐known estimator of *τ* ^2^; the RE between‐study variance component as $\tau^{2}=\left(Q-\mathrm{df}\right)/\left[\sum_{j=1}^{M}w_{j}-\left(\sum_{j=1}^{M}w_{j}^{2}/\sum_{j=1}^{M}w_{j}\right)\right]$. DerSimonian and Laird [17] and Schwarzer et al. [18], among others, have provided interpretations of these statistics and alternatives.

A RE model weights each study by the inverse of the sampling variance plus a constant that represents the variability across the main effects, whereas a FE model weights each study by the inverse of the sampling variance. We rerun the analyses with a new weight: *w*_j_* = 1/(SE_*j*_^2^ + *τ* ^2^). A RE model has the FE model as a special case when *τ* ^2^ = 0. A 100 × (1 − *α*)% CI is generally formed with a normal distribution: $\bar{\mathrm{ES}}\pm z_{\alpha /2}\;\sqrt{{\hat{V}}^{2}\left(\bar{\mathrm{ES}}\right)}$. *Q* and *I* ^2^ might be computed from the RE model or for bivariate versions [18, 19].

Here, Δ*C*_*j*_ and Δ*E*_*j*_ can serve as ES_*j*_, for the numerator and the denominator of the ICER separately. SE_*j*_ may be reported in the original CEA paper or derived from other statistics reported; for example, SE = (upper limit of CI − lower limit of CI)/(1.96 × 2) for the standard normal distribution or other divisor for the *t*‐distribution. If SE_j_ is available in some studies, not all, Scenario 1 methods can be used, or a subset of complete SE information or imputation might be utilized under Scenario 2, with discretion.

Under both scenarios, different types of CIs that account for the *bivariate* nature of the denominator and the numerator in ICER can be constructed [18, 20, 21]. Here, we simply use univariate CI for the denominator and the numerator separately, due to the challenges in interpreting the ICER at different CE quadrants/regions mentioned earlier and less importance of accounting for multiplicity and correlation between cost and effectiveness.

In summary, after computing the pooled ICER (from the pooled cost difference and the pooled effectiveness difference) under Scenario 1 or 2, we can overlay the pooled ICER and 95% CI box (with 95% univariate CIs for cost and effectiveness) or ellipse [22].

### 2.3 How to Compute 95% CI/Arc of the ICER

We explain how to construct a 95% CI (strictly speaking, arc) of the ICER in this subsection. Numerical values of confidence limits can be difficult to understand; they are best understood graphically on the CE plane. We adapted a Bootstrap “wedge” method proposed by Obenchain [23, 24]. Let us assume that we have *M* studies available with required input data:

- Step 1 (how to prepare for the CE plane): Sample *M* studies by simple random sampling *with* replacement, the effectiveness–cost pair as correlated observations within each study.
- Step 2: Compute {Δ*E*, Δ*C*, ICER} with *M* studies using a (weighting) method in Section 2.
- Step 3: Repeat Steps 1 and 2 *B* times (e.g., *B* = 500 or 1000), to generate *B* sets of bootstrap replicates and {Δ*E*, ΔC, ICER}.
- Step 4: Plot each pair of values for {Δ*E*, Δ*C*} in the CE plane; then ICER = Δ*C*/Δ*E* is the slope.
- Step 5 (how to compute a 95% CI/arc for ICER):
  – If bootstrap replicates are in one, two, or three quadrants: the locations of the two tails are apparent, so we sort the B ICER values and select the 2.5th and 97.5th percentiles for a 95% CI/arc; one in the clockwise direction (clockwise bound [CB]) and another in the counterclockwise direction (counterclockwise bound [CCB]). When we sort, we must use “reordering” when we hit ±∞ or discontinuity in the middle.
  – If bootstrap replicates are in all four quadrants: this can happen when effect difference and cost difference are both close to 0; do “reordering” of the ICER replicates first as needed (i.e., we should sort bootstrapped ICERs within each quadrant then conjoin adjacent quadrants correctly), and do inward counting; select the 47.5th and 52.5th percentiles as confidence limits, which is equivalent to 47.5% clockwise and 47.5% counterclockwise cutoffs (so contain central 95%), for a 95% CI/arc of the ICER.

For example, natural order (e.g., smaller to larger, negative to positive) is still valid if all bootstrap replicates are in NE only or NE/SE. On the other hand, if bootstrap replicates are in three quadrants NW/NE/SE, reordering is necessary based on location (between NW and NE, but not between NE and SE), not just numerical values; for example, correctly ordered as (−1, −5, …, −97, −1000, 9999, 864, …, 2, 1, −1, −50, …). Also, four quadrants generally imply high uncertainty, or a CI/arc is not informative to decision‐making. In a simplest one‐quadrant scenario, the proposed bootstrap wedge method reduces to the standard bootstrap percentile method.

Alternatively, traditional Taylor, Fieller, and classical bootstrap methods can be considered, which have long been studied for ICER, with extensive simulations and applications available [25, 26, 27]. For instance, we can set *Y*_*j*_ = *N*_*j*_Δ*C*_*j*_ and *Z*_*j*_ = *N*_*j*_Δ*E*_*j*_ as in Section 2.1 and proceed with the standard ratio method. Yet, traditional methods may not handle different quadrant scenarios well or uniformly. We anticipate that the bootstrap wedge method will not perform well when few studies are to be combined (say, < 5) due to many ties expected in the bootstrapped ICER values. In contrast, Taylor, Fieller, and standard bootstrap methods may not handle three‐ or four‐quadrant scenarios correctly. In each CI result, we should always indicate quadrant locations and numeric values together, as illustrated in our real examples.

## 3 Data Analyses

**FIGURE 1:**
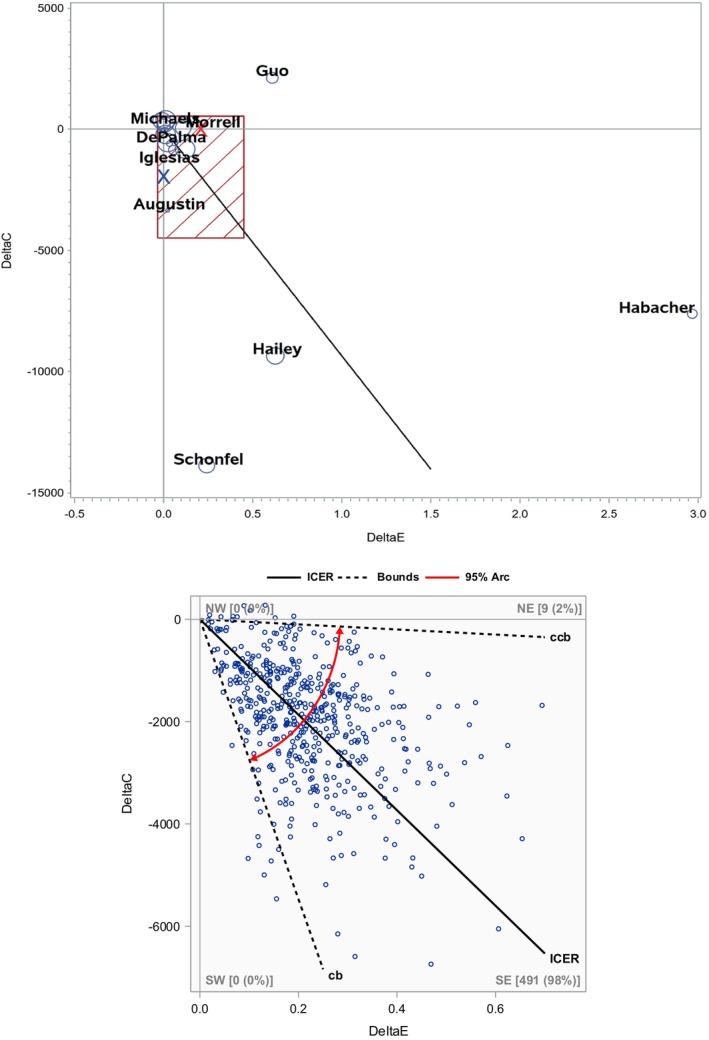
Real examples—Tricco study. *Upper figure*: Rectangle is a 95% confidence interval box for the effect and cost. *X* indicates the point estimate. Size of the bubble is proportional to the study size (i.e., sample size). Solid line represents the summary incremental cost‐effectiveness ratio (ICER). *Lower figure*: 95% confidence interval/arc for ICER. CB denotes clockwise bound, and CCB denotes counterclockwise bound. Dots represent 500 bootstrap replicates. Note that the ranges of axes are different in the upper and lower figures. See Table 1 for original data and Table 2 for numeric results.

**FIGURE 2:**
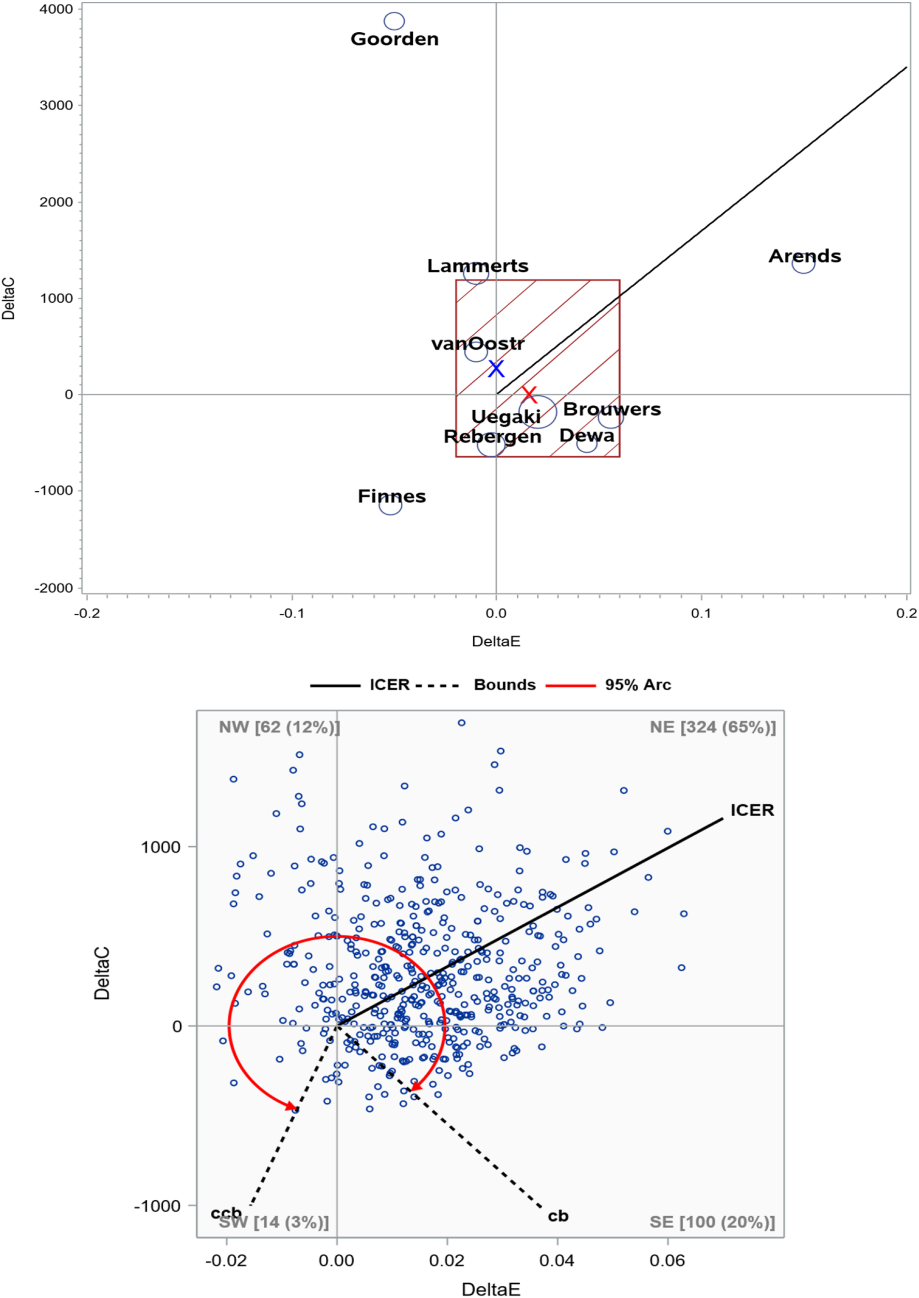
Real examples—Dewa study.

In this section, we provide the two exemplary meta‐analyses of published systematic (literature) reviews. The original authors did not conduct a meta‐analysis. Of note, our illustrations should be viewed as “statistical demonstration or exercise” as we did not conduct a critical appraisal of “combinability” and other nonstatistical issues that are beyond the scope of our paper/proposal; rigorous analysis intended for clinical or economic conclusions should be done together with health economists and subject matter experts (e.g., on comparability or appropriate conversion of currency, years and time horizon, regions, types of effectiveness measures, and PICO). For rigorous data (e.g., currency) conversion, adjustment, and harmonization, check Bagepally et al. [6]. Here, we used a commonly considered classical WTP of $50 000 as a CE threshold for QALY, while other thresholds such as $100 000 or higher, or 3 × Gross Domestic Product can be utilized [2, 3, 28]. Our meta‐analyses are presented in the CE plane in Figures 1 and 2, the data are presented in Table 1, and the numerical results are summarized in Table 2. We propose a “CE plane” as a means to convey meta‐analysis results graphically for the CEA, similar to the famous “Forest plot” for the (standard) meta‐analysis of the EA. We noted the limitations of the Forest plot in the CE meta‐analysis. The size of the bubble on our CE plane can be proportional to the study size, which can be the sample size of the study. Secondary/sensitivity analyses and sample SAS and Excel codes are provided in Supporting Information S2 and S3.

**TABLE 1:** Sample data.

**A. Tricco data**				
Author	DeltaC	DeltaE	*N*	Effectiveness measure used in original publications
Augustin	−3362	0.025	25	Ulcer‐free week
DePalma	−601	0.033	38	Ulcer‐free week
Iglesias	−213	0.01	434	QALY
Iglesias	−566	0.02	387	QALY
Michaels	183	0.0002	213	QALY
Morrell	44	0.11	233	Ulcer‐free week
Watson	371	−0.009	337	QALY
Pham	395	0.009	424	QALY
Schonfeld	−13 883	0.24	240	Ulcer‐free month
Guest	−835	0.054	83	QALY
Dumville	195	0.011	267	QALY
Guo	2137	0.609	126	QALY
Habacher	−7625	2.97	119	Patient year
Jansen	−822	0.12	402	Lifetime QALY
Hailey	−9337	0.63	305	QALY
Xakellis	−25	0.005	39	Ulcer‐free day

**B. Dewa data**						
Author	DeltaC	DeltaE	*N*	SE of DeltaC	SE of DeltaE	Effectiveness measure
Brouwers	−234	0.056	194	.	.	QALY
Rebergen	−520	−0.0027	240	235	0.03	Sick leave days^a^
Uegaki	−184	0.02	433	763	0.018	QALY
Goorden	3874	−0.05	126	750	0.028	QALY
Finnes	−1141	−0.052	177	16 364	.	QALY
vanOostrom	443	−0.01	145	539	0.026	QALY
Arends	1358	0.15	158	1488	0.072	Days to recurrence
Lammerts	1254	−0.01	186	.	0.036	QALY
Dewa	−503	0.044	126	.	0.022	Days lost^a^

*Note:* Dot (.) denotes missing information. Data were retrieved and converted (as needed) from Tricco et al. [9] and Dewa et al. [10]. Data in Dewa et al. are not standardized. DeltaC, DeltaE, and SE are estimates. The data provided here are for statistical analyses, not for rigorous economic or clinical interpretations.

Abbreviations: N, sample size; QALY, quality‐adjusted life year; SE, standard error.

^a^ As a larger value implies a worse outcome, we changed the sign in DeltaE before pooling.

**TABLE 2:** Data analysis results.

Number of studies, scenario, and parameter	Point estimate (95% CI or arc)
Original study: Tricco study	
M = 16, Scenario 1	
Cost difference (in 2013 USD)	−1973 (−4492, 547) using Ratio
	(−4689, 744) using Ratio‐constant
Effectiveness difference (in QALY)	0.21 (−0.03, 0.45) using Ratio
	(−0.25, 0.67) using Ratio‐constant
ICER	−9332 (CCB = −498, CB = −27 354 both in SE)
CE summary, number of studies in each quadrant	NE: 5, NW: 1, SE: 10, SW: 0
Original study: Dewa study	
*M* = 9, Scenario 1	
Cost difference (in original scale)	272 (−646, 1189) using Ratio
	(−1009, 1552) using Ratio‐constant
Effectiveness difference (in QALY)	0.016 (−0.02, 0.06) using Ratio
	(−0.04, 0.07) using Ratio‐constant
ICER	16 548 (CCB = 63 966, 0 in SW) U (0, −∞ in NW) U (∞, 0 in NE) U (0, CB = −27 338 in SW)^a^
CE summary, number of studies in each quadrant	NE: 1, NW: 3, SE: 3, SW: 2

*Note:* In all analyses, the Ratio methods were used because the standard error was not always available. See Table S1 for sensitivity or re‐analyses.

Abbreviations: CB, clockwise bound; CCB, counterclockwise bound; CE, cost‐effectiveness; CI, confidence interval; ICER, incremental cost‐effectiveness ratio; M, number of studies; NE‐NW‐SE‐SW, Northeast‐Northwest‐Southeast‐Southwest; QALY, quality‐adjusted life year; USD, United States dollar.

^a^ For easier understanding of confidence CI/arc for the ICER, see CE planes in Figures 1 and 2.

### 3.1 Example 1: Wound Interventions

Complex wounds impose substantial economic burdens on healthcare systems and patients. A systematic review of CEA of complex wound interventions and care management was published to help identify optimal treatments for specific wound types [9]. Original authors summarized key characteristics and outcomes of CEA for different types of ulcers: years of original publication and of cost assessment; country of original currency; perspective of CEA; study design; sample size; time frame; and funding source. These were tabulated and stratified by ulcer type. Treatment and outcomes‐related data retrieved from original studies include: study treatment and comparator; ICER (non‐numeric) summary or estimate; unit of effectiveness; and incremental cost and effectiveness. Variability measure of SE or CI was not reported in this systematic review, partly because they were not commonly reported in original publications; these conditions fit Scenario 1.

In our analysis, we included studies irrespective of ulcer type, with effectiveness measured in time scales (year, month, or day), including QALY, lifetime QALY, patient‐year, and ulcer‐free year gained (excluding rate, percent, or count outcomes). As mentioned above, these clinical outcomes may not always be combinable from clinical or health economic perspectives, but we combined them for a “statistical” application. Cost data were already converted to 2013 USD in the systematic review. ICER estimates were reported in some studies, but a non‐numeric summary was more commonly provided (e.g., dominant, dominated, which quadrants) [24]. The data from 16 studies included in our analyses are tabulated in Table 1, and the analysis results are displayed in Figure 1 and Table 2.

The pooled cost difference among 16 studies is −$1973 (95% CI: −4492, 547) in 2013 USD, and the pooled effectiveness was 0.20 (−0.03, 0.45) years in QALY using the Ratio method, while Ratio‐Constant yielded (−4689, 744) and (−0.25, 0.67), respectively. Based on these pooled estimates, the ICER estimate is −$9332/year (−27 354, −498) in SE. Among 16 individual ICER values, 10 are in the SE quadrant (dominant), 5 in NE (and 4 under WTP), 1 in NW (dominated), and 0 in SW [29]. Therefore, cost‐effectiveness as well as effectiveness of active interventions when compared to control interventions are apparent (e.g., 14/16 = 88% of studies in cost‐effective regions/quadrants), although the null value 0 is still included in the 95% CIs for cost as well as effectiveness. Bootstrap‐based 95% CI/arc also supported the same tentative conclusion, for example, 98% of bootstrap replicates lie in the SE quadrant (i.e., the study intervention is dominating). Of note, bootstrap replicates lie in NE/SE, so reordering was not needed, so that essentially a standard percentile method works. After excluding one outlier in effectiveness, the results were quite robust; see Table S1. The corresponding CE plane, along with the confidence ellipse, is presented in Figure S1.

### 3.2 Example 2: Return‐to‐Work Interventions Related to Mental Illness

Dewa et al. [10] published a systematic literature review to evaluate the CE of return‐to‐work interventions for mental illness‐related sickness absence. As descriptors of studies, country, interventions, study population (including sample size), design, and data timepoints, the CE perspective and effectiveness outcomes were summarized. Results of CEA, incremental cost and incremental effectiveness, ICER (or its variants such as incremental cost utility ratio), and/or summary of results presented in the CE plane (mostly, % in each of the four quadrants) were documented in the systematic review. As in the previous example, we focused on the studies based on “time (in years)” as the effectiveness measure (six out of nine studies reported QALY; one used sick leave days; one used time to recurrence; and one used time lost); we changed the sign when a larger value implies a worse outcome. The cost unit was not standardized to a common currency in a specific year, and we analyzed these data as they were reported. Seven out of nine studies are from the Netherlands, one from Sweden, and one from Canada; rigorous analyses should employ cost conversion to the most meaningful standard.

If we use the methods for Scenario 1, we could include nine studies in the meta‐analysis. If we use the method for Scenario 2, we could include five studies. The data analyzed are available in Table 1, where some SEs were derived from CIs. Meta‐analyses are presented in Figure 2 and Table 2. The pooled cost difference among nine studies was $272 (95% CI: −646, 1189), and the pooled effectiveness was 0.02 (−0.02, 0.06) years under Scenario 1 using Ratio. Similar to Example 1 and simulation results, Ratio‐Constant provided wider CIs. Based on these pooled estimates, the ICER estimate is $16 548/year. Among nine individual ICER values, one study is placed in the NE quadrant (and smaller than the WTP threshold), three in SE, three in NE, and two in SW. Thus, the decision on CE is far from unanimous, while the overall ICER (< WTP) and raw data points seem to support weakly that study interventions might be cost‐effective. Bootstrap‐based CI/arc also confirmed high uncertainty; replicates lie on all four quadrants with a very wide angle in CI/arc in the around‐the‐clock shape.

When we applied the Scenario 2 method, we could only include five studies as SE is not available in all nine studies. For comparison and illustration only, the methods suited for Scenarios 1 and 2 were applied to five studies, and the results are summarized in Table S2. In practice, Scenario 1 may be preferable in this situation, as a majority of meta‐analysts and readers may wish for the maximum number of qualified studies to be combined. The pooled cost difference and effectiveness difference among five studies and resulting ICERs based on the different methods varied markedly, partly due to a near 0 value in the denominator. Random and Constant *N*_*j*_ produced qualitatively comparable CIs, where conservative CIs by the latter were observed again. Similarly, a wider CI was observed for IVW‐RE than FE, which has long been reported. The primary and re‐analyses might point to study interventions being potentially cost‐effective with high uncertainty. Thus, compared to the previous wound intervention example, the return‐to‐work example revealed that CE is less definitive, so more evidence and discussion are needed before drawing a conclusion. We also note that the null value (0,0) is closer to the center of the 95% CI or box compared to the first example, and the denominator is close to 0.

High heterogeneity was apparent, especially for cost: *Q* = 8.9 and *I* ^2^ = 0.55 for effectiveness and *Q* = 33 and *I* ^2^ = 0.88 for cost with *M* = 5 analyses. High heterogeneity was also reported in INB‐based meta‐analyses, which could also be influenced by varying WTP thresholds, where the SE of the INB is a function of the WTP [6, 7].

### 3.3 Example 3: Simulated Data

We conducted a simulation study and reported the descriptions and summary of results in Supporting Information S1. Note that our simulations are for the “mean” (assuming SE is available) using the four estimators. When SE is not available, only ratio methods are viable options.

## 4 Discussion

Due to the extreme popularity of meta‐analyses, statistical methodologies, and medical publications for treatment effect meta‐analyses have flourished, and worldwide practice guidelines and statistical software are well established. Systematic review and meta‐analysis are often performed together in EA. In stark contrast, CE meta‐analyses are nearly absent due to their secondary nature as well as methodological complexities. Especially controversial is how to pool ICER values, partly because the numerator and denominator should be preserved along with information conveyed in two‐dimensional quadrants. Also, with a near 0 value in the denominator (i.e., when two treatments yield very similar clinical outcomes), a tiny change in the denominator can make ICER = −∞ to ∞. Moreover, the standard meta‐analytic method for a 1‐dimensional parameter, INB, is straightforward, but it conveys inherent problems, for example, necessitating WTPs that can vary from study to study or by context. With this background, systematic reviews have been performed on cost‐effectiveness but did not move on to meta‐analysis to summarize CEA or ICERs. Systematic review is important and should always be considered after a sufficient number of papers have been published, but evidence synthesis cannot be offered, or it can be crude (e.g., based on visual inspection or counting); toward quantitative empirical evidence *synthesis*, meta‐analysis is generally needed.

We proposed simple methods that transfer naturally from a traditional framework so that meta‐analytic methods for CEA and EA could be reasonably consistent and straightforward in implementation. As is typical in meta‐analyses (only with summary statistics available from published papers without IPD), our method (for Scenario 2) requires: cost difference in mean; effectiveness difference in mean; and associated SEs, which are a direct extension of standard meta‐analysis for EA. Another method (Scenario 1) we suggested allows more relaxed data availability: we only need the cost difference in mean, the effectiveness difference in mean, and the total sample size for each study, so SE information is not required. As SE is not routinely reported or derivable, or not always well defined (e.g., discontinuity, ∞ within CI, or flipped order [25, 26, 27]) in the CEA context, a scenario/method in addition to one that is a direct extension from the mainstream methods based on IVW could enhance the feasibility of meta‐analysis on CEA. We computed a CI for cost and effectiveness separately using a standard method, while the quadrant‐based bootstrap wedge method was used for ICER CI/arc. Our method can handle all possible scenarios in a *unified* framework, regardless of which and how many quadrants are involved; standard or adapted Taylor or Fieller methods can be limited but still useful for specific quadrant scenarios [23, 24]. Five decision regions in the CE plane could further assist decision making [3, 23]. The methods we proposed here are based on Taylor approximation, cluster sampling, and weighted estimator (e.g., IVW, a linear combination), so they are easy and intuitive to understand the underlying mathematical and operating mechanisms and to program and implement them. We provide basic Excel and SAS codes, which can be easily improved or adapted.

In this study, we chose to study methods to summarize the ICER because it is still the most widely accepted CEA measure despite well‐documented methodologic challenges [3, 12]. Similar decisions were made regarding the use of QALY, WTP threshold, and CE plane (instead of the popular forest plot). We intentionally avoided variable transformation (and back‐transformation) or other central tendency measures, such as median, following the current practice guidelines in health economics and policy [2, 3, 13, 23]. Focusing on summarizing the evidence separately for the numerator and the denominator of the ICER avoids challenges from combining two different studies with two identical ICERs that have two different meanings (e.g., a new treatment is $100 cheaper but people live one less year vs. a new treatment is $100 more expensive and people live one more year; same ICERs, different meanings).

Importantly, Shields and Elvidge [4] listed the 10 challenges in synthesizing CE estimates. These 10 points should be consulted before planning or performing any meta‐analysis for CEA; this checklist can even be incorporated into the study protocol and reporting guidelines of CEA meta‐analysis in the future, similarly to PROSPERO (International Prospective Register of Systematic Reviews) and PRISMA (Preferred Reporting Items for Systematic Reviews and Meta‐Analyses), which are nearly mandatory in the systematic review and meta‐analysis. As such, their qualitative guidelines and our statistical methods can be complementary. Meta‐analysis should not be conducted when it is not well justified. The same maxim can be true for EA, although there is a lesser concern, and extensive research and discussions have been made on EA over several decades. As Shields and Elvidge [4] and Bagepally et al. [6] explained, it is imperative for meta‐analysts for CEA to be familiar with the additional challenges, which are not generally relevant to meta‐analysts for EA. Particularly, the comparability/conversion of cost across studies is critical or even a prerequisite, along with issues related to study duration or time horizon for CEA, discounting rates, choice of control treatment (beyond placebo, which is common in RCT but may not be meaningful in real‐world settings), among others. Standardization can also be an issue for EA meta‐analysis (e.g., MD vs. SMD).

Additionally, readers may review controversies around IVW‐based methods, particularly regarding the predominant status of the RE model with the DerSimonian‐Laird estimator for *τ* ^2^ as a *default* [14, 18, 30]. The primary advantages of the ratio estimator lie in avoiding the need for within‐study SEs, but also in the possibility of a better‐behaved finite‐sample distribution; being model‐free; and use of minimum variance unbiased estimators with easy estimation of necessary estimates. Ratio versus IVW methods may have different target parameters. When the within‐study sample sizes are large, the IVW estimators have the usual first‐order asymptotic properties under standard regularity assumptions, including smoothness in the parameters. These regularity conditions are typically satisfied in cost or effectiveness estimation problems, and that is a reason why IVW estimators have been used by many practitioners. In situations where the within‐study sample sizes are small, the large sample asymptotic theory might not apply, and the inference for IVW estimators could be less reliable.

Future developments and refinement may address IPD, INB, bivariate nature, subgroup analysis, meta‐regression, publication bias, heterogeneity, and combinability, and better CIs [17]. Of note, we did not aim to identify a best method as our proposal could serve as an initial prototype for developing greater discussion on methods for this task. For instance, there are recommendations on how to choose FE versus RE in the literature (without full consensus), and RE is much more popular, almost as a norm [18, 30]. Our simulations showed IVW‐RE's suboptimal performance under some settings [31]. Future methods and guidance may provide more rigorous or advanced approaches and advice on implementations. Most likely in practice, the number of studies available for CEA meta‐analysis will be much smaller than the number for EA. Furthermore, publication or selection bias can be more likely for CEA, partly because meta‐analysis for EA should be registered and inclusion criteria for meta‐analysis for CEA are more stringent, with less strict rules on WTP and inherent uncertainty, variability, skewness, reporting convention, and subjectivity in CEA in general, compared to EA. Cost data are much more difficult to access, but all meta‐analysts should always check clinical trial registries—along with other standard sources for meta‐analysis, including existing systematic reviews—to minimize omitted studies and selection bias. Our simulation study can be somewhat simplistic, still reporting initial observations. Extensive simulation studies were conducted for IVW‐based and competing methods for normal and non‐normal data [32]. The *t*‐distribution might not provide a good small sample approximation for incremental cost, incremental benefit, or ICER. It requires validation in simulations, while it works well in meta‐analysis of low‐event binomial trials [33]. Also, constant versus random sample size/weight can be an interesting topic in theory as well as simulation.

In summary, our methods can facilitate meta‐analysis for CEA in a principled manner similar to EA. The two scenarios and methods we suggested can accommodate different data availability and assumptions, where one scenario could allow maximal sample sizes and numbers of studies for inclusion due to minimal input requirements (i.e., only means and sample size), and the other scenario follows the traditional meta‐analysis framework (so that meta‐analysis for EA and CEA can be more consistent). The final result can be summarized in the CE plane—a classical tool in CEA—and statistical inference may be attempted. Contemporary statistical software is highly standardized or even nearly automated for meta‐analysis of EA. In the future, CEA meta‐analysis may follow a similar path, but ideally with more consideration and training for methodological as well as nonstatistical issues in mind.

## Funding

This work was supported by the National Institutes of Health (UL1 TR001860).

## Conflicts of Interest

The authors declare no conflicts of interest.

## Supporting information


**Supplement 1** Supporting Information.
**Figure S1:** Re‐analyses of Tricco study in Figure 1.
**Table S1:** Re‐analyses of Table 2.
**Table S2:** Simulation.

**Supplement 2** Sample SAS code for the analyses of Dewa study.

**Supplement 3** Sample Excel code.

## Data Availability

Data and sample SAS and Excel codes are provided in a table and Supporting Information S2 and S3. All programs for simulation and data analyses were submitted to the journal website and can also be available from the first author upon request.
