# Supplementary material for: Meta‐Analysis of Cost‐Effectiveness
Source: Stat Med. 2026 Mar 18;45(6-7):e70352. doi: 10.1002/sim.70352 (PMC12999371; doi:10.1002/sim.70352)
Supplement: Supplementary file 1 — Supplement 1 Supporting Information. Figure S1: Re‐analyses of Tricco study in Figure 1. Table S1: Re‐analyses of Table 2. Table S2: Simulation. [file SIM-45-0-s002.docx]

**Supplementary Materials**

Figure S1. Re-analyses of Tricco study in Figure 1

A. Excluding one outlier


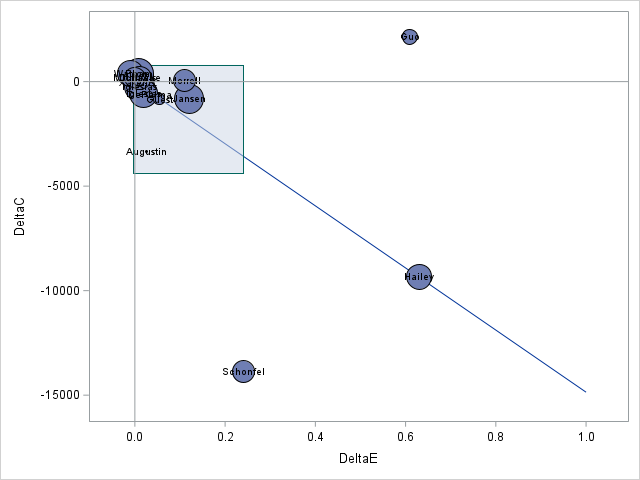


B. Using confidence ellipse in place of confidence box


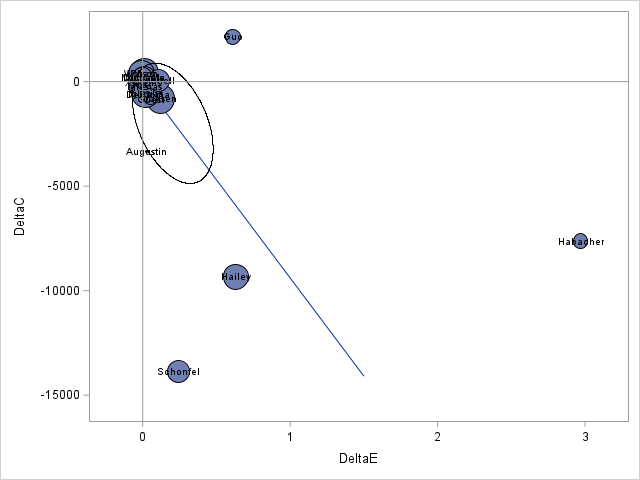


A confidence ellipse accounting for bivariate nature may be considered via the following formulas for the pooled ΔC and ΔE (Nixon et al., 2010; Pepe 2004):

ΔC=sqrt(-2log(1-α)) σ_ΔC_ cos(Ɵ-arccos(ρ_ΔC,ΔE_)/2)+μ_ΔC_,

ΔE=sqrt(-2log(1-α)) σ_ΔE_ cos(Ɵ+arccos(ρ_ΔC,ΔE_)/2)+μ_ΔE_

for Ɵ ϵ [0, 2π).

Of note, normally, we draw the ellipse of confidence intervals for bivariate variables based on their correlations, and the direction of the long axes can be calculated using eigenvalue decomposition. It can be different from the direction of the ratio measure ICER.

Table S1. Re-analyses of Table 2

| Scenario and Parameter | Point estimate (95% CI) |
| --- | --- |
| Original study: Tricco study (M=15, excluding 1 outlier) | |
| **Scenario 1; Ratio** | |
| Cost difference | -1783 (-4367, 800) using Taylor  (-4519, 953) using Constant |
| Effectiveness  difference | 0.12 (-0.004, 0.24) using Taylor  (-0.02, 0.26) using Constant |
| ICER | -14858 |
| CE summary,  number of studies in each quadrant | NE:5, NW:1, SE:9, SW:0 |
| Original study: Dewa study (M=5 with SE available) | |
| **Scenario 2; IVW - Fixed effect** | |
| Cost difference | -27.7 (-416, 360) |
| Effectiveness  difference | 0.001 (-0.02, 0.02) |
| ICER | -27700 |
| **Scenario 2; IVW - Random effect** | |
| Cost difference | 896 (-632, 2425) |
| Effectiveness  difference | 0.001 (-0.04, 0.04) |
| ICER | 896000 |
| **Scenario 1; Ratio** | |
| Cost difference | 510 (-1398, 2418) using Taylor  (-1948, 2969) using Constant |
| Effectiveness  difference | 0.02 (-0.05, 0.10) using Taylor  (-0.08, 0.13) using Constant |
| ICER | 25500 |
| CE summary,  number of studies in each quadrant | NE:1, NW:2, SE:1, SW:1 |

Abbreviations: CE, cost-effectiveness; CI, confidence interval; ICER, incremental cost-effectiveness ratio; IVW, inverse-variance weighting; M, number of studies; NE-NW-SE-SW, Northeast-Northwest-Southeast-Southwest; QALY, quality-adjusted life year; SE, standard error; USD, United States dollar.

These analyses are for demonstration of statistical methods implementation and comparison, not for clinical or economic conclusion.

Table S2. Simulation

| Setting &  Method | M | N | Bias (%) | SSE/ESE | MSE | 95% CI width  (mean/median) | CP  (%) |
| --- | --- | --- | --- | --- | --- | --- | --- |
| 1. Asymmetric effect size (skewness ≈ 2), Truth = 20 | | | | | | | |
| Ratio  Ratio – Constant  IVW – FE  IVW – RE | 50 | 30 | 0.04  0.04  -3.08  -2.45 | 0.42/0.40  0.42/0.42  0.48/0.38  0.47/0.43 | 0.18  0.18  0.60  0.46 | 2.39/1.06  1.68/1.09  1.21/0.89  1.39/1.04 | 97  98  37  56 |
| Ratio  Ratio – Constant  IVW – FE  IVW – RE | 20 | 30 | -0.08  -0.08  -2.94  -2.43 | 0.62/0.52  0.62/0.55  0.60/0.49  0.60/0.55 | 0.38  0.38  0.70  0.59 | 3.90/1.57  2.51/1.65  1.62/1.28  1.88/1.46 | 98  95  64  80 |
| Ratio  Ratio – Constant  IVW – FE  IVW – RE | 10 | 30 | -0.14  -0.14  -2.99  -2.30 | 0.74/0.66  0.74/0.68  0.73/0.63  0.71/0.72 | 0.54  0.54  0.89  0.71 | 5.61/2.58  3.46/2.66  2.37/2.15  2.67/2.40 | 95  93  78  89 |
| Ratio  Ratio – Constant  IVW – FE  IVW – RE | 5 | 30 | -0.08  -0.08  -2.53  -1.78 | 0.90/0.83  0.90/0.85  0.93/0.79  0.89/0.93 | 0.81  0.81  1.11  0.92 | 9.31/4.85  5.53/4.09  3.03/2.88  3.54/3.37 | 96  95  82  94 |
| Ratio  Ratio – Constant  IVW – FE  IVW – RE | 30 | 100 | -0.08  -0.08  -1.03  -0.87 | 0.27/0.30  0.27/0.30  0.28/0.24  0.26/0.31 | 0.07  0.07  0.12  0.10 | 1.93/0.77  1.22/0.78  0.90/0.74  0.98/0.77 | 92  92  80  85 |
| Ratio  Ratio – Constant  IVW – FE  IVW – RE | 20 | 100 | -0.14  -0.14  -1.07  -0.88 | 0.31/0.31  0.31/0.32  0.32/0.28  0.30/0.34 | 0.09  0.09  0.15  0.12 | 2.42/0.88  1.40/0.89  0.98/0.81  1.11/0.86 | 94  94  88  95 |
| Ratio  Ratio – Constant  IVW – FE  IVW – RE | 5 | 500 | 0.01  0.01  -0.14  -0.09 | 0.23/0.22  0.23/0.22  0.24/0.21  0.23/0.24 | 0.05  0.05  0.06  0.05 | 2.57/1.33  1.24/0.94  0.81/0.78  0.90/0.86 | 95  92  90  92 |
| Ratio  Ratio – Constant  IVW – FE  IVW – RE | 5* | 20,30, 50,100, 1000* | 0.01  0.01  -0.38  -0.84 | 0.27/0.18  0.27/1.24  0.28/0.29  0.39/0.47 | 0.07  0.07  0.08  0.18 | 1.02/0.93  6.43/5.75  1.12/1.12  1.71/1.38 | 85  >99  94  96 |
| 1. Asymmetric effect size (skewness ≈ 1), Truth = 30 | | | | | | | |
| Ratio  Ratio – Constant  IVW – FE  IVW – RE | 50 | 30 | 0.25  0.25  -0.52  -0.43 | 0.35/0.30  0.35/0.31  0.36/0.31  0.35/0.33 | 0.12  0.12  0.15  0.14 | 1.39/0.95  1.23/0.96  1.08/0.89  1.14/0.96 | 93  94  86  93 |
| Ratio  Ratio – Constant  IVW – FE  IVW – RE | 20 | 30 | -0.06  -0.06  -0.93  -0.75 | 0.43/0.55  0.43/0.57  0.46/0.46  0.44/0.58 | 0.19  0.19  0.29  0.24 | 4.01/1.30  2.48/1.34  1.65/1.16  1.87/1.31 | 95  96  88  94 |
| Ratio  Ratio – Constant  IVW – FE  IVW – RE | 5 | 30 | -0.21  -0.21  -0.89  -0.73 | 0.76/0.73  0.76/0.74  0.80/0.71  0.77/0.82 | 0.57  0.57  0.71  0.64 | 7.42/3.82  4.28/3.36  2.68/2.46  3.11/2.99 | 93  93  90  94 |
| Ratio  Ratio – Constant  IVW – FE  IVW – RE | 20 | 100 | -0.03  -0.03  -0.27  -0.23 | 0.23/0.24  0.23/0.24  0.25/0.26  0.24/0.29 | 0.05  0.05  0.07  0.06 | 1.77/0.73  1.12/0.73  0.91/0.66  0.98/0.73 | 97  97  95  98 |
| Ratio  Ratio – Constant  IVW – FE  IVW – RE | 5 | 500 | -0.12  -0.12  -0.17  -0.16 | 0.18/0.19  0.18/0.19  0.18/0.19  0.18/0.21 | 0.03  0.03  0.03  0.03 | 2.21/0.88  1.10/0.81  0.69/0.63  0.79/0.70 | 97  95  95  96 |
| Ratio  Ratio – Constant  IVW – FE  IVW – RE | 5* | 20,30, 50,100, 1000* | -0.06  -0.06  -0.15  -0.36 | 0.26/0.16  0.26/1.08  0.26/0.26  0.41/0.41 | 0.06  0.06  0.07  0.18 | 0.89/0.74  5.57/5.70  1.00/1.00  1.51/1.05 | 85  >99  95  97 |
| 1. Asymmetric effect size (skewness ≈ 2), Truth = 20, over-dispersed or bimodal N | | | | | | | |
| Ratio  Ratio – Constant  IVW – FE  IVW – RE | 20 | 30 over-dispersed | 0.42  0.42  -2.90  -2.05 | 0.57/0.54  0.57/0.62  0.69/0.51  0.61/0.61 | 0.32  0.32  0.81  0.54 | 3.40/1.51  2.75/1.74  1.78/1.31  2.09/1.54 | 94  94  54  79 |
| Ratio  Ratio – Constant  IVW – FE  IVW – RE | 20 | 30 or 300  with 50:50% | 0.11  0.11  -0.49  -0.55 | 0.24/0.21  0.24/0.46  0.25/0.21  0.27/0.28 | 0.06  0.06  0.07  0.08 | 1.25/0.64  2.03/1.42  0.72/0.60  0.82/0.71 | 96  99  84  87 |
| Ratio  Ratio – Constant  IVW – FE  IVW – RE | 5 | 30 or 300  with 50:50% | -0.12  -0.12  -0.56  -0.68 | 0.32/0.29  0.32/0.71  0.34/0.33  0.39/0.43 | 0.10  0.10  0.13  0.17 | 3.26/1.46  3.58/2.98  1.20/1.19  1.47/1.36 | 97  98  93  97 |
| 1. Symmetric effect size, Truth = 10 | | | | | | | |
| Ratio  Ratio – Constant  IVW – FE  IVW – RE | 20 | 30 | -0.42  -0.42  -0.20  -0.24 | 0.50/0.47  0.50/0.48  0.50/0.49  0.49/0.53 | 0.24  0.24  0.24  0.24 | 2.78/1.64  2.11/1.74  1.87/1.52  2.08/1.66 | 97  97  95  97 |
| Ratio  Ratio – Constant  IVW – FE  IVW – RE | 5 | 30 | -1.03  -1.03  -1.02  -0.96 | 0.78/0.82  0.78/0.83  0.79/0.83  0.79/0.95 | 0.62  0.62  0.64  0.62 | 9.69/4.23  4.86/3.90  3.12/2.92  3.43/3.19 | 97  95  94  95 |
| Ratio  Ratio – Constant  IVW – FE  IVW – RE | 5 | 500 | 0.64  0.64  0.64  0.64 | 0.22/0.22  0.22/0.22  0.22/0.21  0.22/0.25 | 0.05  0.05  0.05  0.05 | 2.53/1.09  1.24/0.94  0.78/0.73  0.89/0.80 | 98  95  96  97 |
| Ratio  Ratio – Constant  IVW – FE  IVW – RE | 5* | 20,30, 50,100, 1000* | -0.05  -0.05  -0.02  -0.12 | 0.27/0.19  0.27/1.26  0.28/0.29  0.42/0.47 | 0.07  0.07  0.08  0.18 | 1.04/0.96  6.61/6.34  1.13/1.12  1.68/1.15 | 86  >99  95  96 |
| 1. Symmetric effect size, Truth = 10, with data generated from RE model | | | | | | | |
| Ratio  Ratio – Constant  IVW – FE  IVW – RE | 20 | 30 | -0.62  -0.63  -0.63  -0.66 | 0.69/0.61  0.69/0.63  0.69/0.53  0.68/0.65 | 0.48  0.48  0.47  0.47 | 4.45/1.78  2.84/1.83  1.86/1.49  2.25/1.70 | 96  94  86  92 |
| Ratio  Ratio – Constant  IVW – FE  IVW – RE | 5 | 30 | -0.36  -0.36  -0.41  -0.42 | 1.00/0.96  1.00/1.00  1.03/0.83  1.03/1.05 | 1.00  1.00  1.05  1.04 | 13.4/5.40  6.22/4.70  3.20/3.03  3.95/3.70 | 96  95  92  94 |
| Ratio  Ratio – Constant  IVW – FE  IVW – RE | 5 | 500 | 0.22  0.22  0.21  0.19 | 0.57/0.51  0.57/0.51  0.57/0.20  0.56/0.51 | 0.32  0.32  0.32  0.32 | 5.90/2.56  2.94/2.37  0.78/0.78  1.80/1.66 | 98  96  62  88 |
| 1. Symmetric effect size, Truth = 10, with summary statistics generated directly | | | | | | | |
| Ratio  Ratio – Constant  IVW – FE  IVW – RE | 20 | 30 | -2.20  -2.20  -2.50  -2.51 | 1.60/1.55  1.60/1.56  1.60/1.55  1.60/1.73 | 2.58  2.58  2.69  2.58 | 9.10/4.82  6.74/4.82  5.59/4.59  6.08/4.82 | 91  91  90  92 |
| Ratio  Ratio – Constant  IVW – FE  IVW – RE | 5 | 500 | 3.55  3.55  3.75  3.53 | 2.35/2.63  2.35/2.64  2.36/0.65  2.35/2.64 | 5.59  5.59  5.66  5.58 | 33.4/17.2  16.0/13.5  2.60/2.53  9.51/8.87 | 98  97  48  90 |
| Ratio  Ratio – Constant  IVW – FE  IVW – RE | 5* | 20,30, 50,100, 1000* | -5.09  -5.09  -5.10  -1.52 | 3.68/1.21  3.68/5.52  3.64/0.91  2.72/2.67 | 14.0  14.0  13.8  7.37 | 6.50/5.32  24.0/20.7  3.56/3.56  8.75/7.55 | 51  96  36  76 |
| 1. Symmetric effect size, Truth = 10, where effect size depends on N | | | | | | | |
| Ratio  Ratio – Constant  IVW – FE  IVW – RE | 20 | 30 | 0.61  0.61  0.55  0.39 | 0.55/0.52  0.55/0.53  0.57/0.52  0.57/0.58 | 0.31  0.31  0.32  0.32 | 4.41/1.67  2.77/1.73  1.86/1.49  2.17/1.66 | 92  94  90  94 |
| Ratio  Ratio – Constant  IVW – FE  IVW – RE | 5 | 30 | 0.43  0.43  0.54  0.35 | 0.85/0.91  0.85/0.93  0.82/0.84  0.85/1.00 | 0.71  0.71  0.67  0.71 | 10.0/6.62  5.68/4.33  3.16/3.00  3.80/3.52 | 97  96  95  95 |
| Ratio  Ratio – Constant  IVW – FE  IVW – RE | 5 | 500 | 1.11  1.11  1.07  0.36 | 1.09/1.09  1.09/1.09  1.09/0.21  1.08/1.09 | 1.18  1.18  1.20  1.17 | 12.4/5.42  6.49/4.88  0.80/0.78  3.90/3.36 | 96  93  35  86 |

Footnote: Abbreviations: CI, confidence interval; CP, coverage probability; ESE, estimated standard error; FE, fixed effect; IVW, inverse-variance weighting; M, (average) number of studies except for * where it is fixed; MSE, mean squared error; N, (average) sample size in a study except for * where sample sizes are fixed; RE, random effect; SSE, sampling standard error.

A total of 500 simulations were used. Bias is relative bias. Raw data were generated from individual participants then summary statistics for each study were derived as meta-analysis inputs. N is generated from a Poisson distribution. Truth is derived from a∙X+b where X=normal distribution with mean 0, Chi-square distribution with degrees of freedom of 10, or exponential distribution with mean 1 for symmetric, low asymmetric (skewness parameter ≈1) and high asymmetric data (skewness parameter ≈2), respectively, where constants a and b were selected to determine scale and location. A negative binomial distribution with mean of N=30 and probability of 0.25 was used for over-dispersed N. For bimodal data, 50% studies had mean of N=30 and 50% had mean of N=300 from Poisson. For the RE model, RE was generated from N(0,1).

For setting F, summary statistics (i.e., effect size, standard error, N) were generated directly, without raw data, which may reflect real world settings, where only summary statistics are available and meta-analysts should use those statistics only without raw data or rigorous data checks. Variance of effect size was generated via chi-square distribution with df=N-1, multiplied by variance and divided by N-1.

For setting G, we generated data from a∙X+b+N/10-E(N)/10, where a∙X+b is same as above and N-E(N) is random variable of sample size, N, with centering by mean to make the true mean value unchanged.

Ratio-Taylor and Constant denote methods for Scenario 1, where Taylor means random N and Constant means non-random N.

In our simulations, we have effect sizes and their standard errors and N from each study. In a common practice where standard errors are not available, only Ratio methods are feasible, not IVW methods. Simulation results may not be necessarily comparable across different simulation settings (also with different seeds) so performance metrics may be compared for the four methods within the same simulation setting. Also this simulation study assumed standard error is available from included studies. If not, only Ratio methods can be usable.

*Description of simulation set-up:* We conducted a simulation study to evaluate numerical performance of the candidate methods for “mean” estimation: let us call them, Ratio-Taylor, Ratio-Constant, IVW-FE, and IVW-RE. We adopted the following evaluation metrics: relative bias (in %); sampling standard error (SSE) vs. estimated standard error (ESE); mean squared error (MSE); length of 95% CI; and coverage probability (CP). We considered the data generation scenarios as follows: 1) ES are skewed vs. symmetric; 2) the number of studies to be pooled, M=5, 10, 20 or 50 on average; 3) sample size of original studies, N=30, 100 or 500 on average; and 4) sample size over-dispersed vs. not. Additionally, we added an extreme scenario: M=5 with N=20, 30, 50, 100, and 1000. We used a 5% alpha level. Sample results under different combinations of simulation configurations are summarized in Table S2. SAS 9.4 was used for data generation as well as analyses (SAS Institute, Cary, NC).

Raw data or individual participant data (IPD) in each study were generated first then summary statistics were computed for each study; for most meta-analyses, only summary statistics are available. Specifically, we generated the number of studies to be pooled, M, from a uniform distribution with a minimum of 3, and N from Poisson or Negative binomial distribution with a minimum of 5, where the latter distribution is for over-dispersion (e.g., probability of 0.25 for N=30). Additionally, we generated bimodal data for N; 50% studies had mean of 30 and 50% had means of 300 from Poisson. Symmetric and asymmetric distributions could be appropriate for effectiveness, whereas asymmetric distribution would be more appropriate for cost. We used a normal distribution for symmetrical data. For asymmetric data, an exponential and chi-square distributions were employed. From all study settings, we only used the following statistics in the final analysis: {ES and N, or ES and SE} from M individual studies, which is typical in standard meta-analysis settings without IPD. We conducted 500 simulations under each simulation scenario. True parameter value was computed analytically as well as via the average of 1000 simulated values. Truth is derived from X*a+b where X=normal distribution with mean 0, Chi-square distribution with df=10, or exponential distribution with mean 1 for symmetry (skewness parameter=0), low skewness (≈1) and high skewness (≈2), respectively, with constants a and b selected to control scale and location. For the RE model, RE, uj, in Eq (2) was generated from N(0, 1).

Additionally, we simulated two ancillary settings under symmetric ES, a basic setting where the 4 estimators perform most ideally. One is when summary statistics (i.e., ES, SE, N) were generated directly, bypassing raw data, which may reflect real world settings, where only summary statistics are available, and meta-analysts should use those statistics only without raw data or rigorous data checks. Variance of effect size was generated via chi-square distribution. Another is that we generated ES that depends on N.

Simulation results may not be necessarily comparable across different simulation settings so performance metrics may be compared for the four methods within the same simulation setting.

*Summary of simulation results:* Observed biases from the 4 methods were mostly small in all cases. As M or N increased, the length of CI decreased as anticipated. With symmetric distribution on ES, all four methods tended to perform well and comparably. IVW-FE under-performed in some scenarios, while IVW-RE improved the low CP of IVW-FE greatly in many cases and their performances improved when skewness was reduced (2 to 1 to 0); see Table S2. As expected, IVW-FE demonstrated efficiency (e.g., the narrowest CI in all settings) confirming the mathematical theory, whereas the ratio methods tended to yield CIs that can be too wide. Notably, when data were very skewed with small N (e.g., 30), a larger M of studies tended to aggravate the coverage, e.g., M=5 can yield more accurate CP than M=50. Overall, IVW-RE yielded a wider CI than the FE counterpart, which has been documented in the existing literature. When low CP was observed for IVW-FE, discrepancy between SSE vs. ESE is apparent; true sampling variance is severely underestimated by variance estimate. Also, suboptimal performance of IVW-based methods was also reported in a different setting (Landsman et al. 2019). MSE tended to be smaller for the Ratio methods. Between Taylor vs. Constant within the Ratio method, no apparent methodological superiority may be concluded. When N has an outlier or severely bimodal, the Ratio-Constant method yielded extremely wide CIs so very high CPs can imply too conservative coverage, not useful CI, or numerical instability. Under some of those scenarios, in contrast, the Ratio-Taylor yielded low CP and the IVW methods demonstrated better performance over the ratio methods. Sometimes, accurate CP, around 95%, greatly improving upon the low CPs from competing methods was achieved at the expense of a very wide CI. Throughout simulations, trade-offs in the length of CI and CP were demonstrated. Increased skewness or variability, and/or over-dispersion in ES yielded increased MSE.

In terms of two ancillary settings, when summary statistics were generated directly, we observed qualitatively similar results overall to when we generated raw data. However, we found that poor performance can happen to all 4 methods, e.g., too low CP or too wide CI. When ES was a function of N, the length of CI can vary substantially over the 4 methods and again IVW-FE's performance can be worst. Naturally, SE increased and CI became wider, compared to when N was not included in the generation of ES random variates.
